# Functional deficiency of vitamin K in hemodialysis patients in Upper Silesia in Poland

**DOI:** 10.1007/s11255-016-1255-6

**Published:** 2016-03-21

**Authors:** Katarzyna Wyskida, Agnieszka Żak-Gołąb, Jarosław Wajda, Dariusz Klein, Joanna Witkowicz, Rafał Ficek, Sylwia Rotkegel, Urszula Spiechowicz, Joanna Kocemba Dyczek, Jarosław Ciepał, Magdalena Olszanecka-Glinianowicz, Andrzej Więcek, Jerzy Chudek

**Affiliations:** Health Promotion and Obesity Management Unit, Department of Pathophysiology, Medical Faculty in Katowice, Medical University of Silesia, Medyków 18, 40-752 Katowice, Poland; Department of Pathophysiology, Medical Faculty in Katowice, Medical University of Silesia, Medyków 18 Street, 40-752 Katowice, Poland; Department of Internal, Autoimmune and Metabolic Diseases, Medical Faculty in Katowice, Medical University of Silesia, Medyków 14, 40-752 Katowice, Poland; Dialysis Center in Rybnik, Regional Specialist Hospital No. 3, Energetyków 46, 44-200 Rybnik, Poland; Dialysis Center in Tychy, Centrum Dializa Sosnowiec, Narcyzów 24, 43-100 Tychy, Poland; Dialysis Center in Pszczyna, Centrum Dializa Sosnowiec, Antesa 11, 43-200 Pszczyna, Poland; Dialysis Center in Siemianowice Śląskie, Nefrolux, Szpitalna 6, 41-100 Siemianowice Śląskie, Poland; Department of Nephrology, Transplantation and Internal Medicine, Medical Faculty in Katowice, Medical University of Silesia, Francuska 20-24, 40-027 Katowice, Poland; Dialysis Center in Katowice, Centrum Dializa Sosnowiec, Medyków 17, 40-752 Katowice, Poland; Dialysis Center in Chorzów, Centrum Dializa Sosnowiec, Strzelców Bytomskich 11, 41-500 Chorzów, Poland; Dialysis Center in Żory, Centrum Dializa Sosnowiec, Dąbrowskiego 20, 44-241 Żory, Poland; Dialysis Center in Wodzisław Śląski, Centrum Dializa Sosnowiec, Leszka 10, 44-313 Wodzisław Śląski, Poland; Dialysis Center in Sosnowiec, Centrum Dializa Sosnowiec, Jabłoniowa 27, 41-200 Sosnowiec, Poland

**Keywords:** Hemodialysis, Nutrition, PIVKA-II, Undercarboxylated MGP, Vitamin K intake

## Abstract

**Purpose:**

Functional vitamin K deficiency (both K_1_ and K_2_) is postulated to be one of the most relevant links between chronic kidney disease and vascular calcification in hemodialysis (HD) patients. Recommended dietary restrictions in HD patients superimposed on diversity of eating habits across the countries may affect the prevalence of functional vitamin K deficiency. The aim of this study was to determine the level of functional vitamin K deficiency and its relation to vitamin K_1_ intake in HD patients in Upper Silesia in Poland.

**Methods:**

Protein-induced vitamin K absence or antagonist-II (PIVKA-II) and undercarboxylated matrix Gla protein (ucMGP) were assessed by ELISA in 153 stable, prevalent HD patients and 20 apparently healthy adults (to establish normal ranges for PIVKA-II and ucMGP). Daily phylloquinone intake was assessed using a food frequency questionnaire.

**Results:**

PIVKA-II and ucMGP levels were increased in 27.5 and 77.1 % of HD patients in comparison with the reference ranges in apparently healthy controls, respectively. In 45 % of cases, the increased PIVKA-II level was explained by insufficient phylloquinone intake for Polish population (recommended intake: >55 μg for women and >65 µg for men). Applying ROC analysis, we showed that vitamin K_1_ intake below 40.2 µg/day was associated with increased PIVKA-II levels. There was no correlation between vitamin K_1_ intake and plasma concentration of ucMGP, or between PIVKA-II and ucMGP.

**Conclusions:**

(1) Functional vitamin K_1_ deficiency is explained by low vitamin K_1_ intake in less than half of HD patients. (2) Undercarboxylated matrix Gla protein level is a poor surrogate for functional vitamin K_1_ deficiency.

## Introduction

Cardiovascular diseases (coronary artery disease, congestive heart failure, arrhythmias or sudden cardiac death) are the main causes of morbidity and mortality in patients with chronic kidney disease (CKD). Increased mortality in hemodialysis (HD) patients often is associated with accelerated atherosclerosis and excessive vascular calcification [1]. Increased risk of the development of cardiovascular calcification in patients with CKD can only partly be explained by the presence of established risk factors such dyslipidemia, hypertension, smoking habit or diabetes [2, 3]. Functional deficiency of proteins involved in the regulation of calcium metabolism is probably a crucial mechanism for this process [2]. A direct link between the decreased availability of vitamin K and vascular calcification has been suggested by numerous studies [4–7]. The term ‘vitamin K’ refers to a group of compounds consisting of the plant form, phylloquinone (vitamin K_1_), the bacterial form, the menaquinones (MK, vitamin K_2_) and a synthetic form, menadione (vitamin K_3_), which is also an intermediate in vitamin K metabolism [8].

Recent studies indicate both low vitamin K intake and functional vitamin K deficiency among patients receiving renal replacement therapy [9–11]. Vitamin K intake is affected by low-potassium and low-phosphorus recommended diet in HD patients. According to Cranenburg et al. [12], mean daily vitamin K_1_ intake in HD patients was 118 μg and mean vitamin K_2_ intake was 21 μg. Most of the studies using HPLC methods showed undetectable or very low concentrations of menaquinones (especially MK-4) in HD patients [12, 13].

Deficiency of vitamin K, either due to diminished intake or the use of coumarin derivatives, results in undercarboxylation of vitamin K-dependent proteins (VKDPs) [14]. This includes several proteins involved in the regulation of the process of calcification—matrix Gla protein (MGP) and osteocalcin (OC) [14, 15]. The optimal daily vitamin K intake required to activate VKDPs has not been determined; however, data from interventional studies on vitamin K supplementation suggest benefits of this approach [7].

MGP is a calcification inhibitor expressed by vascular smooth muscle cells in the vasculature. To obtain MGP function—inhibition of bone morphogenetic protein 2 (BMP-2), γ-carboxylation of its five residues with the presence of vitamin K as a co-factor is required [16]. The degree of carboxylation required for MGP function is not known. The uncarboxylated form of MGP (ucMGP) does not inhibit the process of vascular calcification; thus, plasma levels of ucMGP reflect the availability of vitamin K in the vessel wall [6]. The accumulation of ucMGP in atherosclerotic lesions and areas of calcification has been reported in several studies [16, 17]. According to Cranenburg et al. [2], the circulating fraction of ucMGP may be decreased in the presence of arterial calcification due to the increased ucMGP accumulation observed in calcified tissues in HD patients; however, the exact mechanism of this process has not been described as yet.

A marker commonly used for the assessment of functional vitamin K (mostly vitamin K_1_) deficiency is the plasma level of protein induced by vitamin K absence or antagonist-II (PIVKA-II). PIVKA-II is a liver-derived VKDP that reflects vitamin K status [2]. According to several recent studies, half of HD patients have subclinical vitamin K deficiency, demonstrated by increased circulating levels of PIVKA-II. Lee et al. [18] showed that 73 % of patients with chronic renal failure had hepatic vitamin K deficiency with elevated PIVKA-II concentrations (>2 ng/mL, mean value 4.48 ng/mL). Recommended dietary restrictions in HD patients superimposed on diversity of eating habits across the countries may affect the prevalence of functional vitamin K deficiency.

The aim of this study was to determine the level of functional vitamin K deficiency and its relation to vitamin K_1_ intake in HD patients in Upper Silesia in Poland.

## Subjects and methods

A total of 153 stable, prevalent HD patients (93 men and 60 women) were included in the study. Patients on HD therapy for less than 6 months, hospitalized patients, patients taking vitamin K antagonists and those with a previous history of gastrointestinal disturbances were excluded from the study. The study protocol was accepted by the local bioethical committee (KNW-2-015/N/3/K). Informed consent was obtained from all individual participants included in the study. The study did not include training with a nutritionist and did not interfere with previous nutritional recommendations.

All HD patients were receiving dialysis three times per week for 3.5 to 5 h (11.7 ± 0.9 h weekly). HD patient characteristics including causes of CKD, duration of HD therapy and *Kt/V* are given in Table 1. The control group consisted of 20 apparently healthy adults (10 men and 10 women) of similar age to the HD patients, with normal kidney function.

**Table 1:** Demographic and clinical characteristics of 153 hemodialysis patients and 20 controls (mean and 95 % CI)

	Hemodialysis	Controls
Subjects characteristics		
Age (years)	62 (59–64)	56 (52–60)
Gender (male/female)	93/60	10/10
Body mass index (kg/m^2^)	26.1 (25.2–26.9)	26.0 (24.5–27.1)
Obesity (BMI ≥ 30 kg/m^2^) (*n*/%)	28/18.3	0
Primary cause of CKD (*n*/%)		
Diabetes	43/28.1	NA
Hypertension	17/11.1	NA
Nephrolithiasis	8/5.2	NA
Autosomal dominant polycystic kidney disease (ADPKD)	10/6.5	NA
Ischemic nephropathy	3/2.0	NA
Glomerulonephritis	24/15.7	NA
Interstitial nephritis	13/8.5	NA
Other or unknown	35/22.9	NA
Co-morbidities (*n*/%)		
Hypertension	138/90.2	0
Diabetes	57/36.3	0
Coronary artery disease	84/54.9	0
Stroke	12/7.8	0
Past kidney transplantation	11/7.2	0
Dialysis parameters		
Time on dialysis (months)	48 (41–56)	NA
Residual diuresis (mL/day)	453 (374–531)	NA
*Kt/V* (per HD session)	1.21 (1.13–1.27)	NA
Ultrafiltration (L/week)	2.5 (2.3–2.6)	NA
Pharmacotherapy (*n*/%)		
*Antihypertensive*	138/90.2	0
No of antihypertensive drugs (*n*)	2.0 (1.8–2.2)	0
Oral anti-diabetic	18/32.7^a^	0
Insulin	37/67.3^a^	0
Antiplatelet	79/51.6	0
Statins	60/39.2	0
Fibrates	0	0
Oral phosphorous binders	127/83.0	0
Carbonate calcium dose (g/day)	3.8 (3.4–4.3)	NA
Sevelamer hydrochloride	4/2.6	0
Cinacalcet	18/11.8	0
Cinacalcet dose (mg/day)	79 (60–98)	NA
Alfacalcidol	18/11.8	0

*NA* non-applicable

^a^For patients with diabetes

The study protocol involved obtaining additional blood samples while performing routine tests (blood count, urea, calcium, phosphate, sodium, potassium) before a midweek HD session and after an overnight fast. Only patients on morning HD sessions were recruited.

### Measurements

Protein-induced vitamin K absence or antagonist-II (PIVKA-II) and ucMGP were assessed by ELISA using commercially available kits (Cusabio, Wuhan, China) with intra-assay and inter-assay coefficients of variability below 8 and 10 %, respectively (for both kits). Detection ranges for PIVKA-II and ucMGP were 0.312–20 and 0.156–10 ng/mL, while the lower limit of detection was 0.078–0.039 ng/mL (according to manufacturer), respectively. For ucMGP determination, 5000-fold dilution was used.

We established the normal ranges for PIVKA-II and ucMGP as the values of the 95 % confidence interval around the mean in 20 apparently healthy adult subjects: 0.37–0.66 ng/mL and 5.1–9.2 mg/mL, respectively.

### Daily phylloquinone intake assessment

Daily phylloquinone, calciferol, calcium, phosphate, sodium, magnesium, iron and potassium, as well as energy and macronutrients intakes (fat, carbohydrates, protein, cholesterol, dietary fiber), were assessed on the basis of a Diet History Questionnaire II (DHQ)—a freely available food frequency questionnaire (FFQ) developed by staff at the Risk Factor Monitoring and Methods Branch (RFMMB). For a purpose of this study, a past year with portion size version of the questionnaire was used. Patients were asked 134 food item and eight dietary supplement past-year intake questions with questions included about portion size. Before receiving the FFQ, each participant was instructed orally about completing the form and printed instructions were also provided. FFQ records were reviewed for completeness.

### Statistical analysis

Statistical analysis was performed with Statistica 10.0 PL Stat Soft Corporation software (www.statsoft.com). The normality of quantitative variables distribution was checked by the Shapiro–Wilk test. Variables with skewed distributions (e.g., vitamin K_1_ intake) were logarithmically transformed for correlation analyses. Results are given as mean values with standard deviations or 95 % confidence intervals (95 % CI), or medians with interquartile ranges. For comparison of groups, we used the *χ*^2^ test (qualitative variables) and ANOVA, followed by Tukey’s test (quantitative variables). The adequacy of statistical power of these analyses was controlled (>0.8). Correlation coefficients were calculated according to Pearson. The receiver operating characteristic (ROC) was used for the establishment of daily K_1_ intake resulting in increased plasma PIVKA-II levels (greater than established reference range for healthy individuals—95 percentile).

Values of *p* < 0.05 were considered to be statistically significant.

## Results

### Plasma concentration of PIVKA-II and ucMGP

The mean plasma concentration of PIVKA-II in HD patients was 0.59 (0.51–0.68) ng/mL (Table 2) and was not significantly different than in healthy subjects—0.51 (0.37–0.66) ng/mL. Increased plasma PIVKA-II concentrations (>0.66 ng/mL) were found in 42 of the HD patients (27.5 %). Additionally, plasma concentration of ucMGP in HD patients was significantly (*p* < 0.001) greater than in healthy subjects [17.9 (16.3–19.5) vs. 7.1 (5.1–9.2) mg/mL]; increased levels (>9.2 mg/mL) were found in 118 of the HD patients (77.1 %).

**Table 2:** Biochemical characteristics of study groups (mean and 95 % CI)

	Hemodialysis	Controls
Hemoglobin (g/dL)	10.7 (10.4–11.0)	14.6 (14.1–15.2)
Total cholesterol (mg/dL)	169 (160–178)	212 (194–229)
LDL cholesterol (mg/dL)	90 (84–95)	142 (125–157)
HDL cholesterol (mg/dL)	28 (26–29)	61 (52–70)
Triglycerides (mg/dL)	159 (142–177)	128 (103–153)
Calcium (mmol/L)	2.14 (2.10–2.19)	Na
Phosphate (mmol/L)	1.77 (1.67–1.87)	Na
Parathyroid hormone (pg/mL)	444 (374–515)	Na
Creatinine (µmol/L)	Na	78 (67–85)
ucMGP (mg/mL)	17.9 (16.3–19.5)	7.1 (5.1–9.2)
ucMGP >9.2 mg/mL (%)	77.1	5.0
PIVKA-II (ng/mL)	0.59 (0.51–0.68)	0.51 (0.37–0.66)
PIVKA-II >0.66 ng/mL (%)	27.5	10.0

*Na* not available

### Daily K_1_ intake

Median (interquartile range) K_1_ intake in HD patients was 103 (43,221) µg (Table 3). No difference was found in vitamin K_1_ intake between men and women. However, the intake was lower than recommended for the Polish population (at least 65 µg/day for men and 55 µg/day for women [15]) in 32 % of HD patients. The intake of vitamin K_1_ was most strongly related to the consumption of protein (*R* = 0.560, *p* < 0.001), fiber (*R* = 0.664, *p* < 0.001) and magnesium (*R* = 0.601, *p* < 0.001). In addition, daily K_1_ intake was proportional to the serum level of HDL cholesterol (*R* = 0.196, *p* < 0.05).

**Table 3:** Energy, macronutrient, micronutrient and vitamin K_1_ intake in 109 HD patients, who returned filled questionnaire (mean and 95 % CI or ^a^median with 25 and 75 percentiles)

	All (*N* = 109)	PIVKA-II ≤0.66 ng/mL (*N* = 75)	PIVKA-II >0.66 ng/mL (*N* = 34)	Statistical significance
Energy intake				
Total (kcal/day)	1639 (1461–1817)	1573 (1378–1768)	1675 (1289–2063)	NS
Macronutrients intake				
Carbohydrates (g/day)	207 (184–229)	198 (175–221)	213 (163–263)	NS
Proteins (g/day)	66 (58–73)	63 (51–82)	67 (51–82)	NS
Fat (g/day)	63 (55–71)	60 (51–70)	63 (47–80)	NS
Fiber (g/1000 kcal)	9.3 (8.8–9.9)	9.3 (8.6–10.1)	9.4 (8.6–10.3)	NS
Micronutrients intake				
Sodium (g/day)	2.92 (2.62–3.22)	2.78 (2.46–3.10)	3.06 (2.41–3.72)	NS
Potassium (mmol/day)	62.9 (56.3–69.5)	60.8 (53.1–68.4)	63.6 (50.3–76.9)	NS
Calcium (mg/day)	591 (519–663)	573 (493–653)	598 (439–742)	NS
Magnesium (mg/day)	224 (202–248)	216 (192–240)	235 (183–286)	NS
Phosphorus (mg/day)	963 (856–1070)	919 (800–1038)	988 (762–1213)	NS
Vitamin K_1_ intake				
Total (µg/day)^a^	103 (43–221)	106 (56–224)	71 (37–203)	NS
Daily intake <55 µg/day in men and <65 µg/day in women (%)	34	27	45	0.08

### Functional vitamin K deficiency and vitamin K_1_ intake

**Fig. 1:**
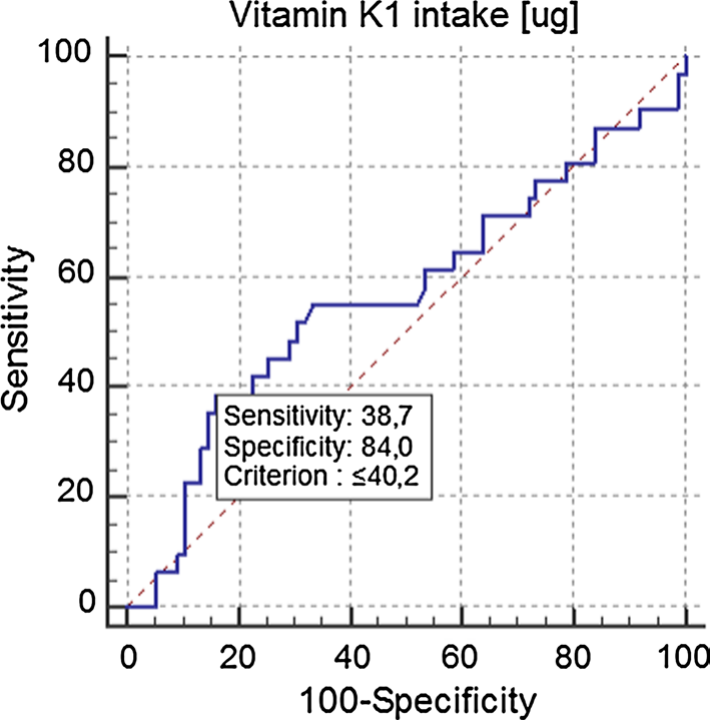
Receiver operator curve analysis showing the threshold daily intake for K_1_ resulting in increased plasma concentration of PIVKA-II (>0.66 ng/mL)

The subgroup of HD patients with increased PIVKA-II levels was characterized by lower daily K_1_ intake (Table 3). The receiver operator curve (ROC) analysis revealed that increased plasma concentration of PIVKA-II was characteristic of participants with a daily K_1_ intake of less than 40.2 µg per day (with 38.7 % sensitivity and 84 % specificity)—Fig. 1.

The second ROC analysis showed that HD patients with daily vitamin K_1_ intake over 98.1 µg/day are characterized by lower risk of increased PIVKA-II levels (with 65.0 % sensitivity and 51.2 % specificity). Approximately 25 % of patients with such an intake had increased PIVKA-II levels.

### Functional vitamin K deficiency and ucMGP levels

The levels of ucMGP were similar in patients with and without functional vitamin K deficiency—17.2 (14.2–20.2) versus 18.4 (16.5–20.3) mg/mL; NS. Additionally, there was no correlation between plasma concentration of ucMGP and PIVKA-II (*R* = −0.016, *p* = 0.85) and between ucMGP and daily K_1_ intake (*R* = 0.028, *p* = 0.77).

## Discussion

The results of our study increase the knowledge concerning the regional variability of the prevalence of functional vitamin K deficiency in HD patients and indicate the need for the standardization of methods used for its assessment.

There are only a few studies assessing vitamin K intake and status in HD patients in the literature [2, 5, 9, 18, 19]. A recent study by Holden and co-workers in 172 subjects with stage 3–5 CKD showed that the criteria for subclinical vitamin K deficiency were met by 6 % of the patients based on circulating K_1_ measurements, by 60 % based on OC carboxylation and by 97 % based on PIVKA-II levels [9]. In a population of 24 HD patients, Lee et al. showed elevated PIVKA-II concentrations in 73 %, while Schlieper et al. described abnormal PIVKA-II concentrations in 64 % of HD patients [5, 18]. In our study, functional vitamin K deficiency was demonstrated by increased plasma PIVKA-II concentrations in 27.5 % of HD patients. Our data are more similar to those obtained by Nerlander et al. [19] who found vitamin K deficiency based upon PIVKA-II measurements in 14.6 % of patients (with 60 % of patients treated with warfarin). The observed variation of the obtained results is mostly related to the shortcomings in the methodology (probably limited specificity of antibodies to recognition uncarboxylated and carboxylated proteins) for functional vitamin K assessment, including measurements of PIVKA-II concentration with commercially available ELISA kits. We cannot exclude that some variation is related to diverse vitamin K intake related to traditional choices of food by specific populations.

In the present study, we utilized an ELISA kit from Cusabio. HD patients with increased plasma concentration of PIVKA-II (>0.66 ng/mL) had markedly lower daily vitamin K_1_ intake (less than 40.2 µg/day) than recommended for the Polish population (>55 μg for women and >65 µg for men). However, among HD patients with increased PIVKA levels, 55 % had adequate dietary vitamin K_1_ intake. Higher daily vitamin K_1_ intake (over 98.1 µg/day) was needed to prevent vitamin K deficiency defined by increased PIVKA-II concentration. In a subgroup with greater intake, increased PIVKA-II levels were observed in a quarter of patients. These data suggest that other factors may also contribute to disturbed vitamin K_1_ metabolism in HD patients, leading to subclinical functional vitamin K_1_ deficiency: e.g., gut microbiota composition, impaired vitamin K absorption or disturbed metabolism. It should be stressed that numerous studies have found adequate vitamin K intake in the majority of HD patients.

According to Cranenburg et al. [12], mean dietary vitamin K_1_ intake among HD patients in the Netherlands was 118 μg/day (18–494) and for vitamin K_2_ was 21 μg/day (2–68). Those data are similar to values obtained in the present study (phylloquinone intake 103 [43–221] µg/day) with 32 % of patients not meeting recommended phylloquinone intake for the Polish population. It should be stressed that the food frequency questionnaire that was used in this study does not allow us to determine vitamin K_2_ intake. We have recently performed a study in 85 HD patients using a three-day food diary that showed mean vitamin K_1_ and MK-4 intake of 98.8 (90–108) and 28.5 (26.2–30.8) μg/day, respectively [13].

Specific diet suggestions for HD patients (low-phosphorus and low-potassium diet) may lead to decreased intake of green vegetables (the main source of phylloquinone) and dairy products (the primary source for menaquinones) that can cause nutritional vitamin K deficiency. However, the compliance with dietary recommendations is usually low.

The process of γ-carboxylation with the participation of vitamin K allows MGP bioactivity to be used as a calcification inhibitor. Our data failed to prove that functional deficiency of vitamin K influences ucMGP levels in HD patients. Unexpectedly, plasma ucMGP concentrations were significantly greater than in healthy subjects (17.9 [16.3–19.5] vs. 7.1 [5.1–9.2] mg/mL; *p* < 0.001). The sparse available published data on ucMGP concentrations in HD patients, published by a single group, are somewhat contradictory results. According to the data from the Heart and Soul Study, decreased serum ucMGP level is associated with reduced glomerular filtration rate [20]. Study by Cranenburg et al. showed markedly lower ucMGP concentrations in HD patients with the lowest values observed in a group with calciphylaxis compared with reference population [2]. In two subsets of HD patients (*N* = 40 and *N* = 120), the same group showed levels of ucMGP in HD patients (193 ± 65 and 173 ± 70 nM/L, respectively) were lower by about 50 % than in apparently healthy subjects of the same age (441 ± 97 and 424 ± 126 nM/L, respectively) [21, 22]. We cannot exclude that the differences found are the consequence of the methodology for ucMGP measurements used by us and by Schurgers’ group.

However, in line with our data, more studies have described elevated concentrations of dephosphorylated-uncarboxylated MGP (dp-ucMGP) in HD patients [5–7, 12, 23, 24]. It should be stressed that only ucMGP and dp-ucMGP could be measured in plasma, and the function of phosphorylation of MGP is, as yet, unknown, though some data indicate that it may play a role in regulating the secretion of proteins into the extracellular environment [25]. We did not determine dp-ucMGP concentrations, and this may be considered to be a limitation of our study.

MGP is produced by vascular smooth muscle cells and is subsequently γ-carboxylated in the presence of vitamin K. It is suggested that this process is more dependent on menaquinone than phylloquinone [16]. In line with this hypothesis, we have shown that ucMGP is not a surrogate marker of functional vitamin K deficiency, as we have observed similar values in a subgroup with normal and increased PIVKA-II levels.

The main limitation of the study was the lack of measurement of phylloquinone levels in serum samples. We did not determine vitamin K_2_ intake due to limitations of the food frequency questionnaire. Additionally, due to a small number of subjects, vitamin K intake in the control group was not assessed, as it may not accurately reflect vitamin K_1_ intake in healthy Polish adult population.

In conclusion, we have shown that functional vitamin K_1_ deficiency is explained by low vitamin K_1_ intake itself in less than half of HD patients and that ucMGP level is a poor surrogate of functional vitamin K_1_ deficiency.
